# Human FAM3C restores memory-based thermotaxis of *Caenorhabditis elegans famp-1/m70.4* loss-of-function mutants

**DOI:** 10.1093/pnasnexus/pgac242

**Published:** 2022-10-25

**Authors:** Masaki Nakano, Ryuki Imamura, Takuma Sugi, Masaki Nishimura

**Affiliations:** Molecular Neuroscience Research Center, Shiga University of Medical Science, Seta-Tsukinowa, Otsu, Shiga 520-2192, Japan; Program of Biomedical Science, Graduate School of Integrated Sciences for Life, Hiroshima University, 3-10-23 Kagamiyama, Higashi-Hiroshima, Hiroshima 739-0046, Japan; Molecular Neuroscience Research Center, Shiga University of Medical Science, Seta-Tsukinowa, Otsu, Shiga 520-2192, Japan; Program of Biomedical Science, Graduate School of Integrated Sciences for Life, Hiroshima University, 3-10-23 Kagamiyama, Higashi-Hiroshima, Hiroshima 739-0046, Japan; Molecular Neuroscience Research Center, Shiga University of Medical Science, Seta-Tsukinowa, Otsu, Shiga 520-2192, Japan

**Keywords:** FAM3C, FAMP-1/M70.4, POMGnT1, *Caenorhabditis elegans*, thermotaxis

## Abstract

The family with sequence similarity 3 (FAM3) superfamily represents a distinct class of signaling molecules that share a characteristic structural feature. Mammalian FAM3 member C (FAM3C) is abundantly expressed in neuronal cells and released from the synaptic vesicle to the extracellular milieu in an activity-dependent manner. However, the neural function of FAM3C has yet to be fully clarified. We found that the protein sequence of human FAM3C is similar to that of the N-terminal tandem domains of *Caenorhabditis elegans* FAMP-1 (formerly named M70.4), which has been recognized as a tentative ortholog of mammalian FAM3 members or protein-*O*-mannose β-1,2-*N*-acetylglucosaminyltransferase 1 (POMGnT1). Missense mutations in the N-terminal domain, named Fam3L2, caused defects in memory-based thermotaxis but not in chemotaxis behaviors; these defects could be restored by AFD neuron-specific exogenous expression of a polypeptide corresponding to the Fam3L2 domain but not that corresponding to the Fam3L1. Moreover, human FAM3C could also rescue defective thermotaxis behavior in *famp-1* mutant worms. An in vitro assay revealed that the Fam3L2 and FAM3C can bind with carbohydrates, similar to the stem domain of POMGnT1. The athermotactic mutations in the Fam3L2 domain caused a partial loss-of-function of FAMP-1, whereas the C-terminal truncation mutations led to more severe neural dysfunction that reduced locomotor activity. Overall, we show that the Fam3L2 domain-dependent function of FAMP-1 in AFD neurons is required for the thermotaxis migration of *C. elegans* and that human FAM3C can act as a substitute for the Fam3L2 domain in thermotaxis behaviors.

Significance Statement Human FAM3C shows sequence homology to the stem domain of protein-O-mannose β-1,2-N-acetylglucosaminyltransferase 1 (POMGnT1) and the N-terminal domains of *Caenorhabditis elegans* FAMP-1; however, functional similarity remains to be clarified. We found that FAMP-1 is required for the memory-based thermotaxis behaviors of *C. elegans*, and missense mutations in the N-terminal Fam3L2 domain of FAMP-1 resulted in defective thermophilic migration. Unexpectedly, exogenous expression of human FAM3C as well as the Fam3L2 domain in AFD thermosensory neurons rescued the defects in thermophilic migration in *famp-1* mutants. Additionally, similar to the stem domain of POMGnT1, the Fam3L2 domain and FAM3C bound to GlcNAc but the thermotaxis-defective Fam3L2 mutants did not. Our findings suggest that these proteins share a common mechanism underlying the functions observed in neuronal cells.

## Introduction

The family with sequence similarity 3 (FAM3) superfamily, composed of FAM3A, FAM3B, FAM3C, and FAM3D, was predicted to share a classical cytokine-like four-helix bundle structure in an earlier study (1). However, recent conformational analyses of FAM3B and FAM3C have revealed that these members adopt a globular β−β−α three-layer architecture with a water-filled cavity (2, 3). The folds of these proteins are very similar to that of the stem domain of protein-O-mannose β-1,2-N-acetylglucosaminyltransferase 1 (POMGnT1) (4). The molecular mechanism underlying the functions of FAM3 members remains unclear; however, POMGnT1 is known to catalyze the transfer of an *N*-acetylglucosamine (GlcNAc) residue to *O*-mannose in glycoproteins. Homozygous mutations of *POMGnT1* lead to defects in the synthesis of *O*-mannosyl glycan and cause muscle–eye–brain disease characterized by congenital muscular dystrophy, ocular abnormalities, and brain malformation with mental retardation (5).

FAM3C shows ubiquitous expression under physiological conditions and plays a role in hepatic gluconeogenesis repression and osteoblast differentiation in mice (6, 7). In addition, FAM3C is involved in epithelial–mesenchymal transition (EMT), carcinogenesis, and metastasis of carcinomas (8–14). Previous studies found that FAM3C signal is received by leukemia inhibitory factor receptor on the cell surface, transduced via the YY1–heat shock factor 1 (HSF-1)–CaM–Akt axis, and executed by several transcription factors including STAT3, HSF-1, and Runx2 (6, 9, 13, 15, 16).

In our previous studies, we have found that FAM3C is widely expressed in neuronal cells of the mammalian brain, including cerebrocortical pyramidal neurons that are fully differentiated and reside in the G_0_ phase of the cell cycle (17–20). Neuronal FAM3C is enriched in presynaptic terminals and released into the extracellular fluid in a synaptic activity-dependent manner (18, 19). Secreted FAM3C binds to presenilins (catalytic components of the membrane-bound γ-secretase complex) and suppresses production of amyloid-β peptides, which play a causative role in the development of Alzheimer’s disease (17). Although the function of FAM3C in the central nervous system (CNS) has been anticipated, a previous study reported that FAM3C-knockout mice exhibited no obvious deficit in basic neuronal functions and survived to adulthood (7). Hence, we investigated the higher function of nervous system such as neuronal plasticity using a simple model organism. Thermotaxis and chemotaxis behaviors of *Caenorhabditis elegans*, of which the underlying neural circuits have been revealed, are considered to be reliable models for studying memory-based migration behaviors (21–23). In this study, we found that FAM3C exhibits significant sequence similarity to the N-terminal tandem domains of *C. elegans* FAMP-1 (formerly named M70.4). Missense mutations in the N-terminal domain of FAMP-1 caused defects in thermotaxis but not in chemotaxis behaviors; these defects could be restored by the AFD neuron-selective expression of this domain. Intriguingly, human FAM3C could also rescue the thermotaxis behavior of *famp-1* mutants. Moreover, an in vitro assay revealed that this domain and FAM3C can bind with carbohydrates, similar to the stem domain of POMGnT1. These results suggest that FAMP-1 is a functional ortholog of mammalian FAM3C.

## Results

### FAM3C-like domains of *C. elegans* FAMP-1

**Fig. 1.**
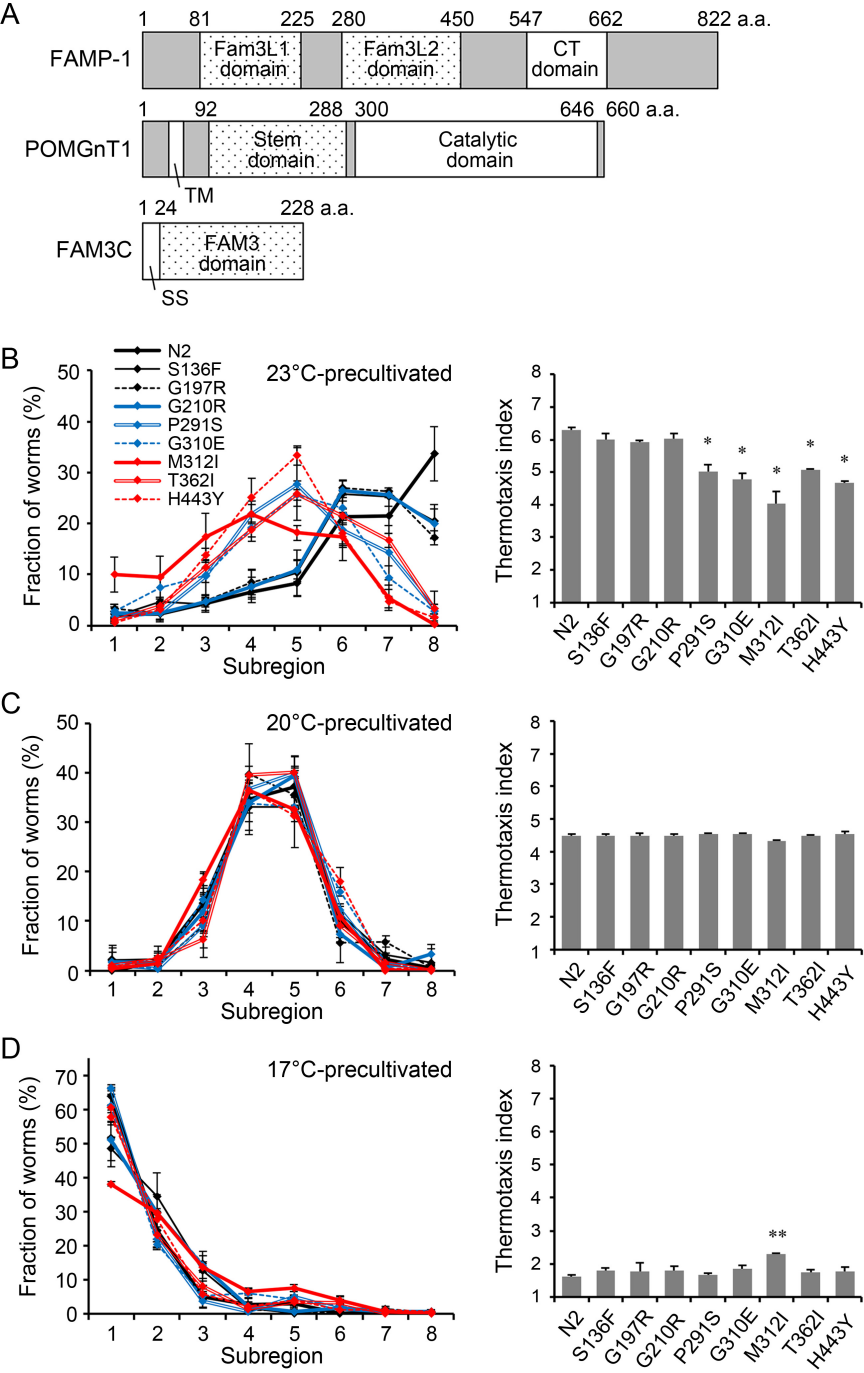
Missense mutations in the Fam3L2 domain of *famp-1* perturbed thermotaxis migration. (A) Schemes of the primary structures of *C. elegans* FAMP-1, human POMGnT1, and FAM3C. CT domain: C-terminal domain; TM: transmembrane domain; and SS: signal sequence. (B)–(D) Thermotaxis migrations of wild-type N2 worms and eight *famp-1* mutants were assessed after precultivation at 23°C (B), 20°C (C), and 17°C (D). Line graphs show the percentage relative distribution of worms in each subregion. Thermotaxis index scores are shown in the bar graphs. Data are means ± SEM; *n* = 3 for each genotype. ** *P* < 0.01, * *P* < 0.05 versus N2 according to ANOVA with Tukey’s post hoc test.

An ortholog search and BLAST against the NCBI nucleotide database provided no *C. elegans* canonical orthologs of FAM3 superfamily members. However, human *FAM3* genes had significant sequence similarity to two coding regions of *famp-1/m70.4* (WBGene00019786) that encode N-terminal tandem domains, here referred to as FAM3-like domains 1 and 2 (Fam3L1 and Fam3L2), respectively (Fig. 1A). Indeed, Fam3L1 and Fam3L2, respectively shared 25% to 29% and 29% to 34% similarity with four members of the human FAM3 superfamily in terms of amino acid sequences (SI Appendix; Figure S1A and B). However, the orthologous relationship among FAMP-1, POMGnT1, and FAM3 members remains obscure because the domain composition is distinct: POMGnT1 has an N-terminal transmembrane domain, a FAM3-like stem domain, and a C-terminal catalytic domain, whereas FAMP-1 has N-terminal duplicated FAM3-like domains and a C-terminal domain (Fig. 1A). Furthermore, the similarity between the catalytic domain of POMGnT1 and the C-terminal domain of FAMP-1 is minimal (SI Appendix; Figure S1C), and it is not yet known whether FAMP-1 exhibits glycosyltransferase activity.

Given that no *C. elegans* canonical ortholog of FAM3 members exists and that FAMP-1 contains duplicated FAM3-like domains, we tested the possibilities of trans-splicing and post-translational endoproteolysis of the FAMP-1 mRNA and protein, respectively. Therefore, we injected the *mec-4* promoter-driven expression plasmid for full-length FAMP-1 into N2 worms, which induced *famp-1* expression in four mechanosensory neurons. Immunoblotting of the worm extract revealed a single band corresponding to the full-length protein with a molecular weight of ∼70 kDa, but no other band of the processed fragment was detected (SI Appendix; Figure S1D); thus, the Fam3L1 and Fam3L2 domains were apparently expressed as parts of the full-length FAMP-1 protein.

### *Defects in the* thermotaxis behaviors *of famp-1* mutants

We obtained all *famp-1* mutant strains that were available from the *Caenorhabditis* genetics center (CGC): VC40639 *famp-1(gk737833)*, VC40642 *famp-1(gk739534)*, VC40848 *famp-1(gk845538)*, VC20615 *famp-1(gk355956)*, VC40532 *famp-1(gk681625)*, VC40952 *famp-1(gk884912)*, VC40352 *famp-1(gk589965)*, VC20300 *famp-1(gk195774)*, VC40447 *famp-1(gk642110)*, VC41013 *famp-1(gk927637)*, and VC20156 *famp-1(gk314930)* strains, harboring S136F, S197R, G210R, P291S, G310E, M312I, T362I, N427I, and H443Y missense and R400X and W445X nonsense *famp-1* gene mutations, respectively (hereinafter, referred to as “mutations”). All mutants showed normal gross morphology. A locomotory rate assay revealed that all mutants, except the N427I mutant and two nonsense mutants, exhibited equivalent locomotor activity to wild-type worms (SI Appendix; Figure S2).

To study the memory-based behaviors of *famp-1* mutants, we employed a thermotaxis assay (21, 22, 24–26). The worms remember the ambient temperature associated with their seeded precultivation conditions, and when placed on an unseeded plate with a spatial temperature gradient, they migrate to the precultivation temperature. Depending on this temperature, the worms move on a thermal gradient toward warmer and colder temperatures (thermophilic and cryophilic migrations, respectively) or stay around the temperature that matches their precultivation temperature (isothermal behavior). To perform population thermotaxis assays, we prepared a device composed of aluminum plates on a Peltier module to provide a stable linear thermogradient ranging from 17 to 23°C. The cultivation surface of each assay plate was divided into eight subregions along the temperature gradient, and the thermotaxis index was calculated in every assay (SI Appendix; Figure S3A and “Materials and Methods” section).

We firstly confirmed that wild-type N2 worms showed thermotaxis behavior as previously described (22, 24, 25). Then, we tested the thermotaxis of eight missense mutants that showed no locomotor deficit. After precultivation at 23°C, five mutants (P291S, G310E, M312I, T362I, and H443Y) showed lower thermotaxis index values than those of wild-type N2 worms (Fig. 1B). The M312I mutant also exhibited weaker cryophilic migration (i.e. had a higher index value) than that of the wild-type, whereas the other mutants had equivalent index values in terms of cryophilic migration and isothermal behavior relative to those of the wild-type (Fig. 1C and D). Athermotactic defect-causing *famp-1* mutations (P291S, G310E, M312I, T362I, and H443Y) were exclusively localized in the Fam3L2 domain (amino acid residues 280 to 450; SI Appendix; Figure S1A), indicating that the Fam3L2 domain-dependent function of FAMP-1 is required for thermophilic migration. In contrast, C-terminal truncation (R400X and W445X) and N427I mutation led to more severe neuronal dysfunction (SI Appendix; Figure S2). We chose the M312I mutant for further analyses, as this mutant exhibited most prominent mutational effects.

### *Fam3L2* rescues *famp-1* mutants from thermotaxis defects

**Fig. 2.**
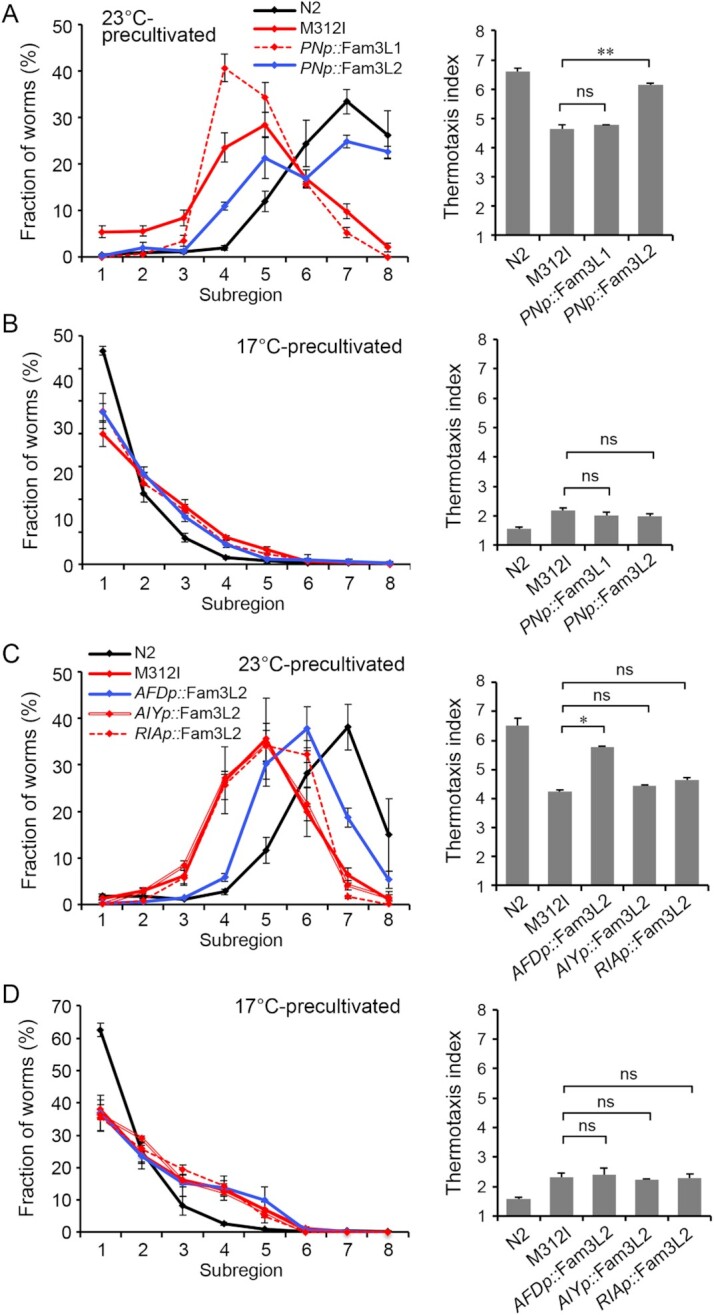
Fam3L2 transfection rescues athermotaxic *famp-1* mutants. (A) and (B) M312I mutant worms were injected with *unc14* promoter-fused cDNA for polypeptides corresponding to Fam3L1 and Fam3L2 (*PNp::* Fam3L1 and *PNp::* Fam3L2, respectively). Thermotaxis migrations of these mutants and wild-type (N2) worms were assessed after precultivation at 23°C (A) or 17°C (B). (C) and (D) M312I-mutant worms were injected with *gcy-18, ttx-3*, and *glr-3* promoter-fused cDNA for polypeptides corresponding to Fam3L2 domains (*AFDp::* Fam3L2, *AIYp::* Fam3L2, and *RIAp::* Fam3L2, respectively). Thermotaxis migrations of these mutants and wild-type (N2) worms were assessed after precultivation at 23°C (C) or 17°C (D). Line graphs show the percentage relative distribution of worms in each subregion. Thermotaxis index scores are shown in the bar graphs. Data are means ± SEM; *n* = 3 for each genotype. ** *P* < 0.01,^*^P < 0.05, and no significant difference (ns) according to ANOVA with Tukey’s post hoc test.

We were unable to assess whether the athermotactic defects of *famp-1* mutants could be rescued by exogenous pan-neuronal expression of full-length FAMP-1 due to the lethal effect of the cDNA injection. However, as shown in Fig. 2(A), we found that *unc-14* promoter-driven pan-neuronal expression of a polypeptide corresponding to Fam3L2 but not Fam3L1 significantly improved thermophilic migration of the M312I mutant. In contrast, Fam3L2 transfection did not have any effect on the cryophilic defect (Fig. 2B). Thus, the missense mutations of the Fam3L2 domain apparently caused a defect in thermophilic migration via the loss-of-function effect in neuronal cells.

The neural circuit underlying thermotaxis behaviors has been revealed (SI Appendix; Figure S3B) (22, 27); ambient temperature is sensed by the AFD and AWC sensory neurons, the inputs of which are transmitted via the AIY and AIY–AIZ interneurons to the RIA premotor neuron, which drives thermophilic and cryophilic migrations, respectively (27, 28). The AFD and AWC neurons also play critical roles in the memorization of cultivation temperature (27). The athermotactic defect observed in the M312I mutant was reminiscent of that caused by ablation or dysfunction of the AFD or RIA neuron (22, 29, 30). Hence, we examined the possibility of neuronal type-specific restoration with the Fam3L2 domain. The *gcy-18* promoter-driven restricted expression of Fam3L2 polypeptides in AFD neurons considerably ameliorated the thermophilic defect in M312I-mutant worms but had no effect on the cryophilic defect, whereas selective Fam3L2 expression in RIA or AIY neurons did not produce a notable alteration (Fig. 2C and D). To rule out the possibility that this rescue was specific to the M312I-mutant, we also examined another *famp-1* mutant with a thermophilic migration defect. Similar results were obtained using H443Y-mutant worms (SI Appendix; Figure S4). Thus, the Fam3L2 domain-dependent FAMP-1 function in AFD neurons is required for intact thermophilic migration.

### *AFD* neurons and the related behaviors of *famp-1* mutants

The endogenous expression of *famp-1* in AFD sensory neurons was revealed in a previous study employing single-cell RNA-sequencing analysis (31). Using M312I-mutant worms carrying a stably integrated *gcy-8p:: gfp* reporter transgene, we investigated the morphology of AFD neurons, finding no obvious abnormalities using fluorescence microscopic analysis (SI Appendix; Figure S5).

In addition to the thermotaxis defect, the disruption of AFD functions caused defects in head avoidance behavior to noxious heat and spontaneous reversal behavior (32, 33). Athermotactic mutant worms and wild-type N2 worms exhibited equivalent withdrawal reactions from local heat stimulation directed at the head via an electronically heated metal tip (SI Appendix; Figure S6). In this assay, the thermal memory formation–defective *crh-1(tz2)* mutant (29) also showed intact avoidance, whereas the thermotransduction–defective (*gcy-8, gcy-18*, and *gcy-23*–triple) mutant exhibited a reduced head avoidance reaction, as previously reported (34).

The reversal frequency of athermotactic mutants and wild-type N2 worms did not differ significantly (SI Appendix; Figure S7). These findings suggest that Fam3L2 mutations (P291S, G310E, M312I, T362I, and H443Y) cause selective deficits in thermotactic navigation among AFD neuron-dependent behaviors.

### *Preserved* chemotaxis of *famp-1* mutants

Given that chemotaxis is another type of *C. elegans* migration behavior, we also performed chemotaxis assays of the *famp-1* mutants as previously described (23). Wild-type and M312I-mutant worms showed equivalent chemotaxis migration behaviors against benzaldehyde, isoamyl alcohol (sensed by the AWC neurons), or diacetyl (sensed by the AWA neurons) odorants (SI Appendix; Figure S8). Therefore, Fam3L2 domain mutations specifically caused deficits in thermotaxis and not chemotaxis migration behavior. This result was consistent with the CeNGEN database (https://www.cengen.org), which shows that *famp-1* is not expressed in the AWC and AWA chemosensory neurons.

### *Egg-laying and* higher temperature escape of *famp-1* mutants

According to the CeNGEN map, *famp-1* is expressed in multiple types of neurons and could be involved in various behaviors such as egg-laying and escape from higher cultivation temperature. The egg-laying muscles receive synaptic input from two classes of neurons, namely the HSN and VC, whereas non-neuronal cells, e.g. vulval muscles, uv1 gland cells, and VulD epithelial cells, are also involved in egg-laying (35). Both the HSN and VC neurons express relatively high levels of *famp-1*. We found that athermotactic *famp-1* mutants retained fewer eggs than wild-type worms, and these deficits could not be rescued via the pan-neuronal expression of Fam3L2 (SI Appendix; Figure S9), suggesting that the egg-laying defect of *famp-1* mutants was attributable to dysfunctions in non-neuronal cells and possibly neuronal cells.

In addition to AFD neurons, FLP sensory neurons in the head and PHC neurons in the tail express higher levels of *famp-1*. To assess thermal nociception via these neurons (34), we tested the mutants’ capacity to escape from higher cultivation temperature. None of the athermotactic *famp-1* mutants showed a deficit in escape reactions from noxious temperatures of 29°C, 31°C, or 33°C, whereas the *gcy-8, gcy-18*, and *gcy-23*-triple mutant exhibited a slight but significant defect in escape from a relatively lower noxious temperature of 29°C (SI Appendix; Figure S10). These results suggest that athermotactic Fam3L2 mutations do not cause dysfunctions in all *famp-1*-expressing neurons.

### *Human FAM3C* substitutes for *Fam3L2 in* thermotaxis restoration

**Fig. 3.**
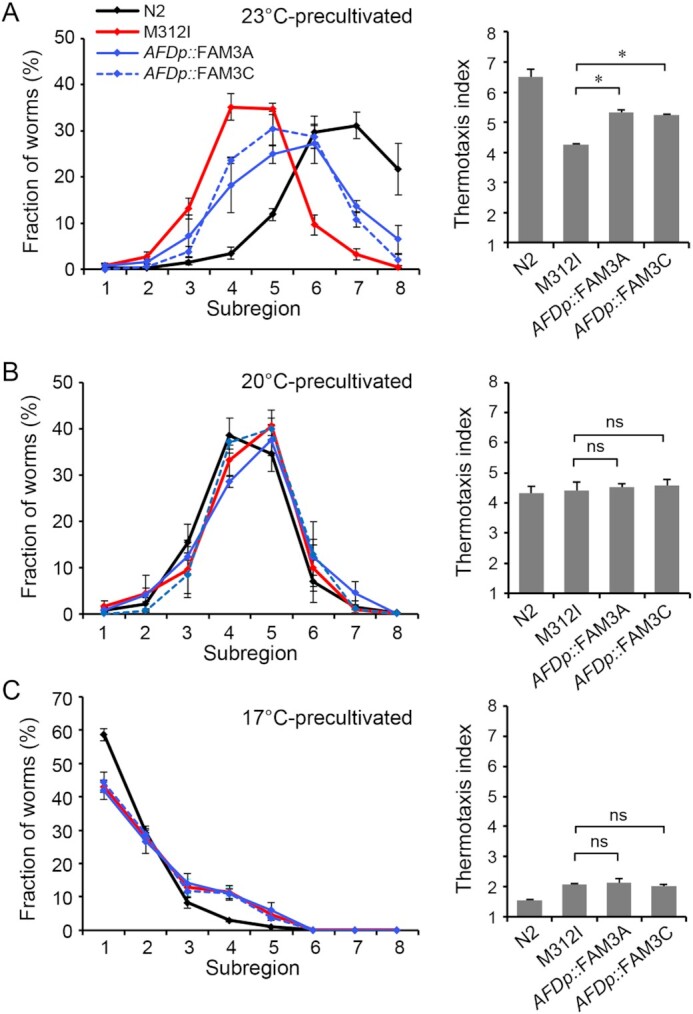
Human FAM3C and FAM3A expression in AFD neurons rescues athermotaxic *famp-1* mutants. (A)–(C) M312I mutant worms were injected with *gcy-18* promoter-fused codon-optimized cDNA for human FAM3C and FAM3A (*AFDp::* FAM3C and *AFDp::* FAM3A, respectively). Thermophilic (A), isothermal (B), and cryophilic (C) migrations of these mutants and wild-type worms (N2) were assessed. Line graphs show the percentage relative distribution of worms in each subregion. Thermotaxis index scores are shown in the bar graphs. Data are means ± SEM; *n* = 3 for each genotype. * *P* < 0.05 and no significant difference (ns) according to ANOVA with Tukey’s post hoc test.

In further tests, we examined whether human FAM3C could restore the thermotaxis behaviors of *famp-1* mutants. AFD neuron-specific expression of codon-optimized human FAM3C partially restored the thermophilic migration of M312I mutants (Fig. 3). Neuronal expression of FAM3C also rescued the thermotaxis defect of H443Y mutants, suggesting that the restoration by FAM3C was not specific to M312I mutants (SI Appendix; Figure S11). Among FAM3 members, FAM3A is also expressed in the mammalian CNS (1, 17, 36); hence, we also investigated potential restoration with human FAM3A, the expression of which resulted in a partial rescue of the thermophilic migration defect in M312I-mutant worms (Fig. 3A).

### Carbohydrate-binding of Fam3L2 and FAM3C

**Fig. 4.**
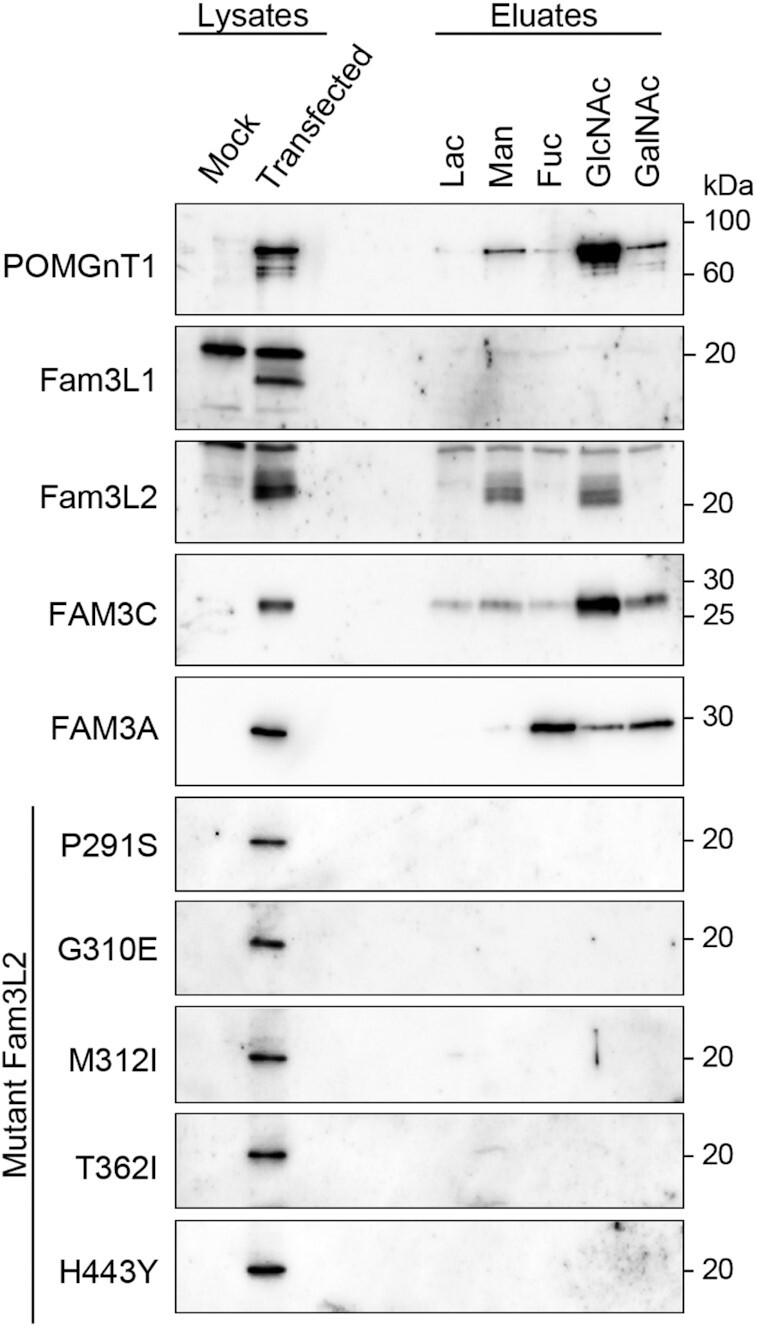
FAM3 members and Fam3L2 domain bind with carbohydrates. Lysates of HEK293 cells transiently transfected with an empty vector (Mock) or expression plasmids for FLAG sequence fused to the stem domain of POMGnT1, codon-optimized Fam3L1 domain, codon-optimized Fam3L2 domain, FAM3C, FAM3A, or mutated Fam3L2 domains were incubated with lactose- (Lac), mannose- (Man), fucose- (Fuc), GlcNAc-, or GalNAc-conjugated resins. The eluates were subjected to immunoblotting with anti-FLAG antibody. Results are representative of three independent experiments.

The stem domain of human POMGnT1 recognizes and binds the β-linked GlcNAc of *O*-mannosyl glycan in which the Arg^129^, Asp^179^, and Arg^207^ residues are directly involved (4). These critical residues are conserved among mammalian POMGnT1, FAM3 superfamily members, and the Fam3L2 domain; however, Asp^179^ and Arg^207^ are not conserved in the Fam3L1 domain (SI Appendix; Figure S1A). Using an in vitro assay to examine carbohydrate-binding ability, we found that the Fam3L2 domain but not the Fam3L1 domain bound to GlcNAc and mannose (Fig. 4). We also found that FAM3C and FAM3A bound to GlcNAc and other carbohydrates (Fig. 4). Additionally, we examined the Fam3L2 domain harboring each athermotactic mutation (P291S, G310E, M312I, T362I, or H443Y). All of these mutations perturbed the binding of the Fam3L2 domain to carbohydrates (Fig. 4). Combined with our findings that Fam3L2, FAM3C, and FAM3A but not Fam3L1 rescue thermotactic defects, these results suggest that the carbohydrate-binding abilities of these proteins and domains might be critical for thermotaxis restoration in *famp-1* mutants.

### Equivalent level of HSF-1 expression in M312I-mutant worms

Previous genetic analysis indicated that the HSF-1-mediated noncell-autonomous signaling was indispensable for thermotactic behavior of *C. elegans*, whereas FAM3C reportedly increased mRNA and protein levels of HSF-1 in the mouse liver (26, 37). These findings suggest that *famp-1* mutations cause thermotactic defects through the downregulation of HSF-1 expression. To test this possibility, we examined the HSF-1 expression levels of the mutant worms. Quantitative real-time PCR indicated no significant difference in HSF-1 mRNA levels between M312I-mutant and wild-type N2 worms (SI Appendix; Figure S12).

## Discussion

Our results indicate the following: (1) sequence similarity exists among FAM3 superfamily proteins, the Fam3L domains of *C. elegans* FAMP-1, and the stem domain of POMGnT1; (2) neuronal FAMP-1 is required for the thermotaxis behaviors of *C. elegans*; (3) missense mutations in the Fam3L2 domain of FAMP-1 cause defects in thermophilic navigation; (4) exogenous expression of the Fam3L2 domain in AFD thermosensory neurons can rescue the athermotactic defect of *famp-1* mutants; (5) Fam3L1 is not functionally redundant with Fam3L2 in thermotaxis behaviors; (6) human FAM3C can substitute for Fam3L2 in restoring the thermotaxis of *famp-1* mutants; and (7) FAM3C and the Fam3L2 domain but not the Fam3L1 and athermotactic mutant Fam3L2 domains bind to GlcNAc. These findings suggest that the Fam3L2 domain-dependent function of FAMP-1 in AFD neurons, which has yet to be clarified, is nonetheless required for the thermophilic migration of *C. elegans*. They also indicate that human FAM3C/A can rescue the loss-of-function of the Fam3L2 domain in thermotaxis behaviors.

The AFD neurons in the bilateral amphid sense organs are the major regulators of memory-based thermotaxis behaviors. Calcium concentration in the AFD cytoplasm preferentially increases in response to warming but not to cooling or unchanged temperature. Thus, an increased cultivation temperature first activates receptor-type guanylyl cyclases, e.g. GCY-8, GCY-18, and GCY-23, followed by cGMP-gated channels composed of TAX-2 and TAX-4, which in turn leads to an influx of Ca^2+^ into AFD neurons (30, 38–41). EAT-4/VGLUT-dependent glutamatergic signals from AFD neurons activate the GLC-3/GluCl inhibitory receptors of AIY interneurons to drive worm migration toward lower temperature (28). PDE-2 and NCS-1 are also critical for cGMP-dependent thermotransduction (42). AFD neurons also play a critical role during the memorization of cultivation temperature (43, 44). Mutations or deletions of several molecules in AFD neurons caused distinct patterns of thermotactic defects. CRH-1 (the ortholog of CREB) mutants showed cryophilic navigation and athermotactic defects after precultivation at 20°C and 23°C, respectively (29). PKC-2 (a Ca^2+^- and diacylglycerol-activated protein kinase C)-depletion resulted in athermotactic behaviors after precultivation at 20°C (45). CEH-14 (the LIM homeobox protein) loss-of-function mutations resulted in athermotactic behaviors in isothermal tracking assays at 20°C (46). RCAN-1 (an inhibitory regulator of TAX-6)-deletion mutants exhibited thermotaxis navigation to a temperature lower than the cultivation temperature (47). INX-4 (a component of the gap junction) mutants exhibited thermotaxis navigation to a temperature slightly higher than the cultivation temperature (48). The epistatic relationship among these genes and the mechanism underlying thermotactic memory formation have yet to be revealed.

The disruption of AFD functions caused multiple behavioral defects, including cultivation temperature-independent athermotactic navigation (22), a robust decrease in the avoidance response to noxious heat (34), and a reduced frequency of spontaneous reversal behavior (33). Among these behaviors, the Fam3L2 mutations selectively caused a defect in thermophilic navigation. Avoidance responses to noxious temperature were affected in the *gcy-8, gcy-18*, and *gcy-23*-triple mutant but not the *crh-1(tz2)* or Fam3L2 mutants, suggesting that Fam3L2 mutations perturbed thermal memory formation rather than thermal sensation in AFD neurons.

Exogenous expression of the Fam3L2 domain or human FAM3C rescued the thermophilic migration of *famp-1* mutants. Fam3L2 shares 34% and 30% similarity with human FAM3C and the stem domain of POMGnT1, respectively (SI Appendix; Figure S1A and B), and FAMP-1 has been recognized as the tentative ortholog of POMGnT1 and/or FAM3 members (49). However, it is difficult to presume the functional mechanism of FAMP-1 and the Fam3L2 domain by analogy with these orthologs. Loss-of-function mutations and gene deletion of *POMGnT1* cause defects in cell–extracellular matrix and cell–cell adhesion that result in developmental abnormalities such as disturbed neuronal distribution and ectopic fibroblasts in the mouse brain (50, 51). In contrast, no abnormalities in the basic functions and gross morphology of the brain were found in FAM3C-knockout mice (7). Mammalian FAM3C is secreted from the presynaptic terminal in an activity-dependent manner, but there is no evidence that FAMP-1 is released from neuronal cells. Our study revealed that FAM3C and the Fam3L2 domain have a carbohydrate-binding ability like that of the stem domain of POMGnT1. However, the functional implications of this finding remain unclear, although the stem domain could be involved in the elongation of *O*-mannosyl glycans (4).

In addition, through our rescue experiments, we were unable to clarify whether Fam3L2 and FAM3C function independently of full-length FAMP-1 or cooperate *in trans* with the other domains of FAMP-1. Notably, however, athermotactic *famp-1*-missense mutations are exclusively localized in the Fam3L2 domain. Moreover, the amino acid residues at these mutations are not conserved in human FAM3C (SI Appendix; Figure S1A). We speculate that the high sensitivity of the Fam3L2 domain to amino acid substitutions is explained by the disruption caused by all mutations to the carbohydrate-binding of this domain.

The orthologous relationship among FAMP-1, POMGnT1, and FAM3 members remains obscure. Many nematode strains have single or duplicated FAM3-like domains, which are always followed by a domain with homology to a part of the C-terminal catalytic domain of POMGnT1 (SI Appendix; Figure S13A). However, nematodes do not have any gene containing an isolated FAM3-like domain. Our database search revealed a gene encoding a protein of a domain structure similar to *C. elegans* FAMP-1 and an absence of FAM3 orthologs in the amphioxus *Branchiostoma floridae* genome. However, such findings were not apparent for the urochordate *Ciona intestinalis*, insect *Drosophila melanogaster*, or other vertebrate genomes. The amphioxus genome sequence exhibits features of the last common ancestor of all chordates, and recent molecular phylogenetic studies position cephalochordates as the basal group within the phylum Chordata, from which urochordates and vertebrates later diverged (SI Appendix; Figure S13B) (52, 53). Hence, FAM3 superfamily orthologs seem to have evolved from the ancient cephalochordates ortholog of a *POMGnT1*-like gene through gene duplication and/or domain shuffling (i.e. they emerged at the very early stage of an ancient chordate lineage).

Several missense mutations in the Fam3L2 domain of FAMP-1 precluded the memory-based thermotaxis behaviors of *C. elegans*, which could be rescued by exogenous expression of human FAM3C as well as the Fam3L2 domain in AFD thermosensory neurons. An in vitro assay indicated that the binding of FAM3C and the Fam3L2 domain to GlcNAc was required for this restoration. Our findings suggest functional similarity of these proteins, sharing a common molecular mechanism mediated through carbohydrate-binding.

## Materials and Methods

### *Caenorhabditis elegans* strains and germline transformation

Wild-type worms (*C. elegans* Bristol N2 strain) were cultivated at 20°C on nematode growth medium plates seeded with *Escherichia coli* OP50-1. The *famp-1* mutants, derived from ethyl methanesulfonate mutagenesis, were obtained from CGC (University of Minnesota) and repeatedly backcrossed to N2 in an attempt to create each isogenic mutant strain. Germline transformation was performed by coinjecting experimental DNA (2 ng/µL) and the coinjection marker pKDK66 (*ges-1p:: nls-egfp*; 50 ng/µL) into the gonad of adult worms (54). Multiple independent transgenic lines were established with each transformation procedure. Transgenic lines expressing *unc-14p*:: Fam3L1, *unc-14p*:: Fam3L2, *gcy-18p*:: Fam3L2-EGFP, *ttx-3p*:: Fam3L2, *glr-3p*:: Fam3L2, *gcy-18p*:: human FAM3C (codon optimized), or *gcy-18p*:: human FAM3A (codon optimized) were obtained from M312I and H443Y mutant worms. Each genotype was confirmed using PCR with specific primer pairs. The *unc-14, gcy-18, ttx-3*, and *glr-3* promoters drove pan-neuronal, AFD neuron-specific, AIY neuron-specific, and RIA neuron-specific expression, respectively (30, 55–57). The *gcy-8, gcy-18, gcy-23*-triple and *crh-1(tz2)* mutant strains were also obtained from CGC.

### Immunoblotting

Worms were rapidly lysed using 30 passes with a Wheaten homogenizer. Immunoblotting was performed as previously described (17). The samples were resolved on NuPAGE 4% to 12% gradient Bis-Tris gels (Life Technologies). Blots were probed using anti-FLAG-M2 antibodies (Sigma-Aldrich).

### Thermotaxis assay

To conduct a population thermotaxis assay, a nematode growth medium plate (13.8 cm × 6.4 cm; 1.9 cm in height) containing 10 mL of thermotaxis medium (3 g/L NaCl, 20 g/L Bacto agar, and 25 mM KPO_4_) was placed on aluminum plates attached to a Peltier thermocontroller. The linear thermal gradient was confirmed using a thermal camera (FLIR; SI Appendix; Figure S2A). Assays were performed at low relative humidity (< 30%). Worms and its progeny were grown on 6-cm plates seeded with *E. coli* OP50-1 at 17, 20, or 23°C until most of the population reached the day 1 adult stage. Once grown, adult worms were washed twice with 2 mL of M9 buffer (3 g/L KH_2_PO_4_, 6 g/L Na_2_HPO_4_, 5 g/L NaCl, and 1 mM MgSO_4_), and approximately 50 to 200 worms were placed at the 20°C region of the gradient. After 60 min, the worms were killed with chloroform gas. The agar surface of the assay plates was divided into eight subregions along the gradient with scores of 1 to 8, and the thermotaxis index was calculated as follows:

$$\begin{equation*}
{\rm{Thermotaxis\ index\ }} = {\rm{\ }}\frac{{\mathop \sum \nolimits_{{\boldsymbol{i\ }} = {\boldsymbol{\ }}1}^{\boldsymbol{N}} {\boldsymbol{i}}\cdot{{\boldsymbol{S}}}_{\boldsymbol{i}}}}{{{{\boldsymbol{N}}}_{{\boldsymbol{total}}}}},
\end{equation*}
$$

where *S_i_* is the number of worms in each subregion and *N_total_* is the total number of worms on an assay plate.

Assays were performed at least three times for each strain or condition.

### Chemotaxis assay

Population chemotaxis assays were performed according to a previously reported method (23). The dilution of attractants was 1:200 for benzaldehyde, 1:100 for isoamyl alcohol, and 1:1,000 for diacetyl with ethanol by volume. Worms were prepared in the way same as was done for thermotaxis assay. The chemotaxis index was calculated using the following formula:

$$\begin{eqnarray*}
&&{\rm{Chemotaxis}}\,{\rm{index}} = ( {\rm{number}}\,{\rm{of}}\,{\rm{worms}}\,{\rm{at}}\,{\rm{attractant}} \nonumber \\
&& \quad - {\rm{number}}\,{\rm{of}}\,{\rm{worms}}\,{\rm{at}}\,{\rm{counterattractant}} )/ \nonumber \\
&& \quad {\rm{total}}\,{\rm{number}}\,{\rm{of}}\,{\rm{worms}}\,{\rm{on}}\,{\rm{an}}\,{\rm{assay}}\,{\rm{plate}}
.
\end{eqnarray*}
$$

### Fluorescence microscopy

PY1322 *oyIs18* worms (58), which expressed the AFD-specific reporter transgene *gcy-8::gfp*, were obtained from CGC and crossed with M312I mutants to identify AFD neurons. L4 worms were anesthetized with sodium azide, immobilized in 1.5% agar between coverslips, and observed using a fluorescence microscope Axio Observer A1 (Zeiss). Images were captured with a CCD camera DP73 (Olympus).

### Head avoidance of noxious temperature

Worms were exposed to noxious heat using an electronically heated metal tip. The temperature at 3.0 mm from the tip of a platinum wire (0.5 mm in diameter) was 33.0°C ± 1.0°C. The assays were performed in a room with a constant temperature of 20°C and a humidity of at least 60%. The heat stimulus was presented in front of the worm, and the initial response was classified according to a previous study (32) as follows: class I, rapid reflexive withdrawal, backing for at least one body length followed by a heading change; class II, rapid reflexive withdrawal but only a little backing; class III, slow backing; class IV, no response. The responses of 100 worms were recorded for each genotype. The data presented represent the percentage of each class.

### Escape from higher cultivation temperature

Adult worms were washed twice, after which approximately 100 to 200 worms were placed on a 6-cm agar plate with food and cultured at 20°C for 60 min. The left half of the surface of the assay plate was then heated to a noxious temperature at 29, 31, or 33°C using a Peltier thermocontroller. Before and 60 min after heating, the number of worms on the right and left surface was counted. Assays were repeated three times for each genotype.

### Assays of body bends and reversal behavior

The frequencies of body bends and spontaneous reversal behavior were evaluated according to the methods in a previous study (33). Well-fed first-day adult worms were transferred from growth plates to standard agar plates with no food. The number of body bends was counted using a standard stereomicroscope. For the assay of reversal behavior, any change from forward to backward movement was scored as a reversal, and these changes were counted for 5 min. In each assay, 20 worms were tested for each genotype.

### Assay of egg-laying behavior

Worms were picked at the early L4 stage and cultured at 20°C before experiments. During the experiment, the worms were allowed to lay eggs for 3 h on a nematode growth medium plate seeded with *E. coli* OP50-1. The number of laid eggs per worm per hour was counted using a standard dissection stereomicroscope.

### Real-time quantitative PCR

Total RNA was extracted from wild-type N2 and M312I-mutant worms using TRIzol reagent (Invitrogen). The 1st strand DNA was synthetized using ReverTra Ace qPCR RT Master Mix with gDNA Remover (TOYOBO). Quantitative PCR was performed on a Light Cycler 480 System II (Roche Diagnostics) using KOD SYBR qPCR Mix (TOYOBO). The following PCR primer pairs were used: Ce-hsf-F and Ce-hsf-R for hsf-1: 5′-CGAAAGATGACTCCACTGTCC-3′ and: 5′-GTCCTCCACAGTTCTTGCC-3′, respectively; Ce-ama-F and Ce-ama-R for ama-1: 5′-TGGAACTCTGGAGTCACACC-3′ and 5′-CATCCTCCTTCATTGAACGG-3′, respectively. The ama-1 mRNA served as an internal control.

### Carbohydrate-binding assay

HEK293 cells were transfected with expression plasmids for Myc-FLAG-tagged human POMGnT1, Myc-FLAG-tagged human FAM3A, 3xFLAG-tagged human FAM3C, FLAG-tagged Fam3L1, and FLAG-tagged Fam3L2 using Lipofectamine LTX (Invitrogen). Extracts of transfected cells were mixed with fucose-, mannose-, lactose-, GlcNAc-, or GalNAc-conjugated resins (CGK-series; EY Laboratories) and incubated at 4°C overnight. Carbohydrate-conjugated resin-bound proteins were eluted using a buffer containing 0.1 M of each free carbohydrate. The eluates were subjected to immunoblotting, and the blots were validated with anti-FLAG antibody (Sigma-Aldrich).

### Statistical analyses

Data are presented as means ± SEM. Statistical analysis was performed using the two-tailed unpaired Student’s *t*-test for two-group comparisons and one-way ANOVA followed by Tukey’s post hoc test for three (or more)-group comparisons. Statistical significance was defined at *P < 0.05 or **P < 0.01.

## Supplementary Material

pgac242_Supplemental_File

## Data Availability

All data are included in the manuscript and/or Supplementary Material.
