# Supplementary material for: Human FAM3C restores memory-based thermotaxis of Caenorhabditis elegans famp-1/m70.4 loss-of-function mutants
Source: PNAS Nexus. 2022 Oct 25;1(5):pgac242. doi: 10.1093/pnasnexus/pgac242 (PMC9802357; doi:10.1093/pnasnexus/pgac242)
Supplement: pgac242_Supplemental_File [file pgac242_supplemental_file.pdf]

Supplementary Information for

Human FAM3C restores memory-based thermotaxis of *Caenorhabditis elegans famp-1/m70.4* loss-of-function mutants

Masaki Nakano[a], Ryuki Imamura[b], Takuma Sugi[a,b]*, and Masaki Nishimura[a]*

[a] Molecular Neuroscience Research Center, Shiga University of Medical Science, Shiga, 520-2192, Japan

[b] Program of Biomedical Science, Graduate School of Integrated Sciences for Life, Hiroshima University, Higashi-Hiroshima, Hiroshima 739-8526, Japan

Correspondence to:

Masaki Nishimura and Takuma Sugi

E-mail: mnishimu@belle.shiga-med.ac.jp.

This PDF file includes:

Figures S1 to S13

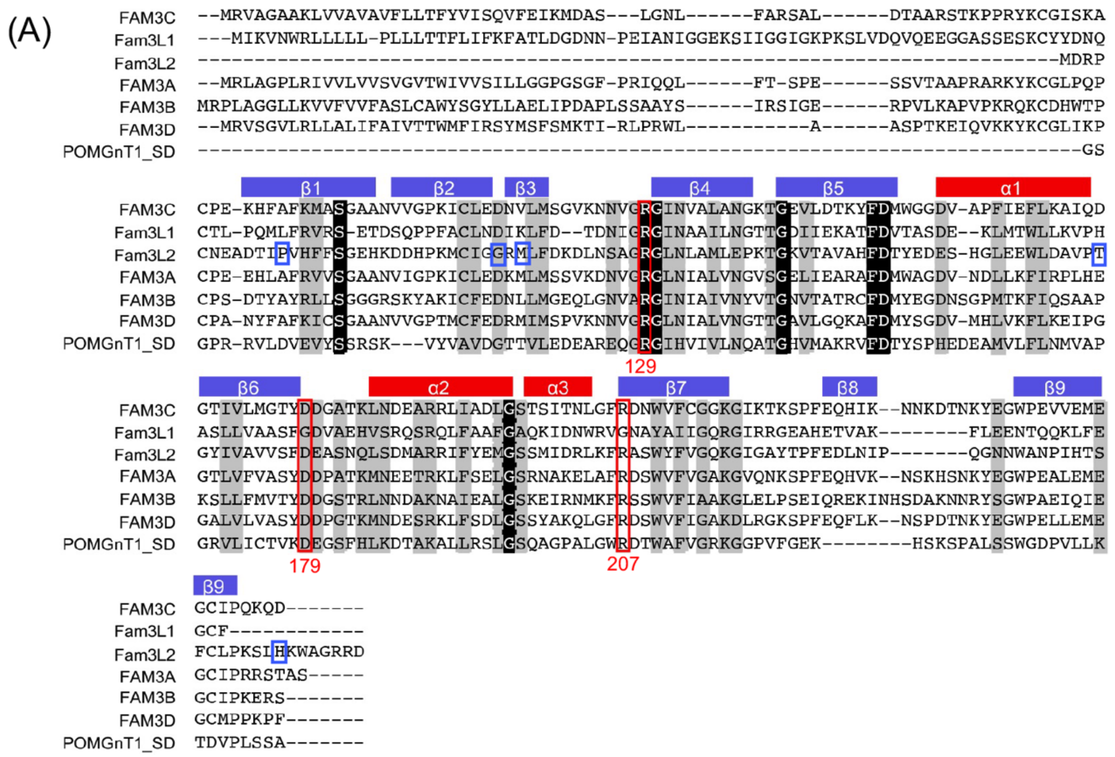

(B)

%	FAM3C	Fam3L1	Fam3L2	FAM3A	FAM3B	FAM3D	POMGnT1
FAM3C	–	26	34	52	37	51	30
Fam3L1	26	–	33	29	25	28	27
Fam3L2	34	33	–	29	29	33	30
FAM3A	52	29	29	–	39	54	27
FAM3B	37	25	29	39	–	33	29
FAM3D	51	28	33	54	33	–	31
POMGnT1	30	27	30	27	29	31	–

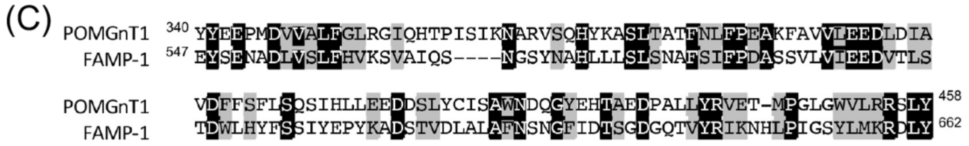

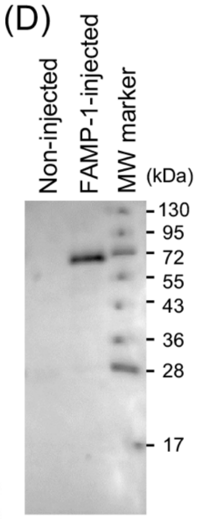

Fig. S1. FAM3 members, *C. elegans* FAMP-1, and POMGnT1

(A) Sequence alignment of human FAM3 members, Fam3L1, Fam3L2, and the stem domain (SD) of POMGnT1. Black and grey boxes highlight identical and homologous residues, respectively. Red and blue bars above the alignment show regions of α-helix and β-sheet, respectively. Red and blue squares indicate residues of carbohydrate-binding and athermotactic mutations, respectively. (B) Percentage of homology among these proteins and domains. (C) Sequence alignment of the catalytic domain of human POMGnT1 and *C. elegans* FAMP-1. Black and grey boxes highlight identical and homologous residues, respectively. (D) Immunoblotting for FAMP-1. Lysates of C-terminally FLAG-tagged FAMP-1 cDNA-injected worms were subjected to immunoblotting with an anti-FLAG antibody.

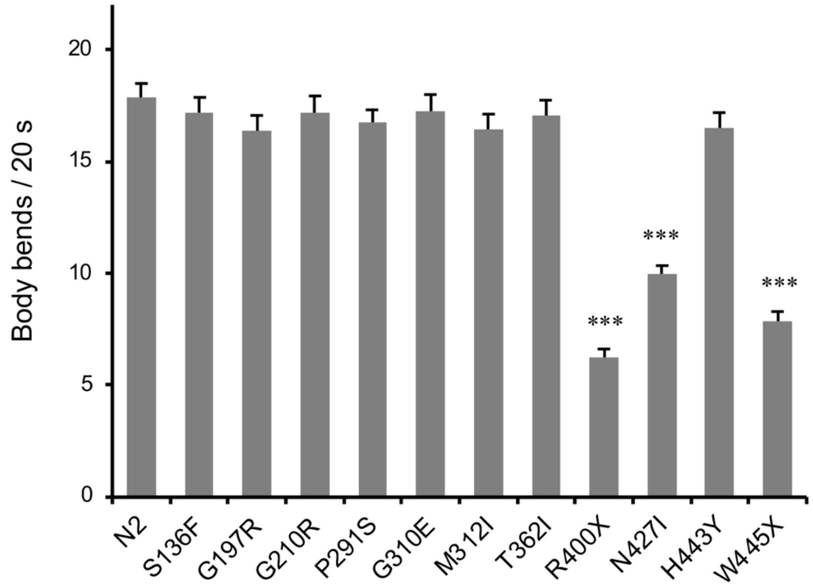

Fig. S2. Body bend assay

Well-fed first day adult worms were transferred from growth plates to standard agar plates with no food. The number of body bends was counted using a standard stereomicroscope. 20 worms were tested for each genotype. Data represent the number of body bends per 20 s per worm (means + SEM). ****P* < 0.001 according to ANOVA with Tukey's *post-hoc* test.

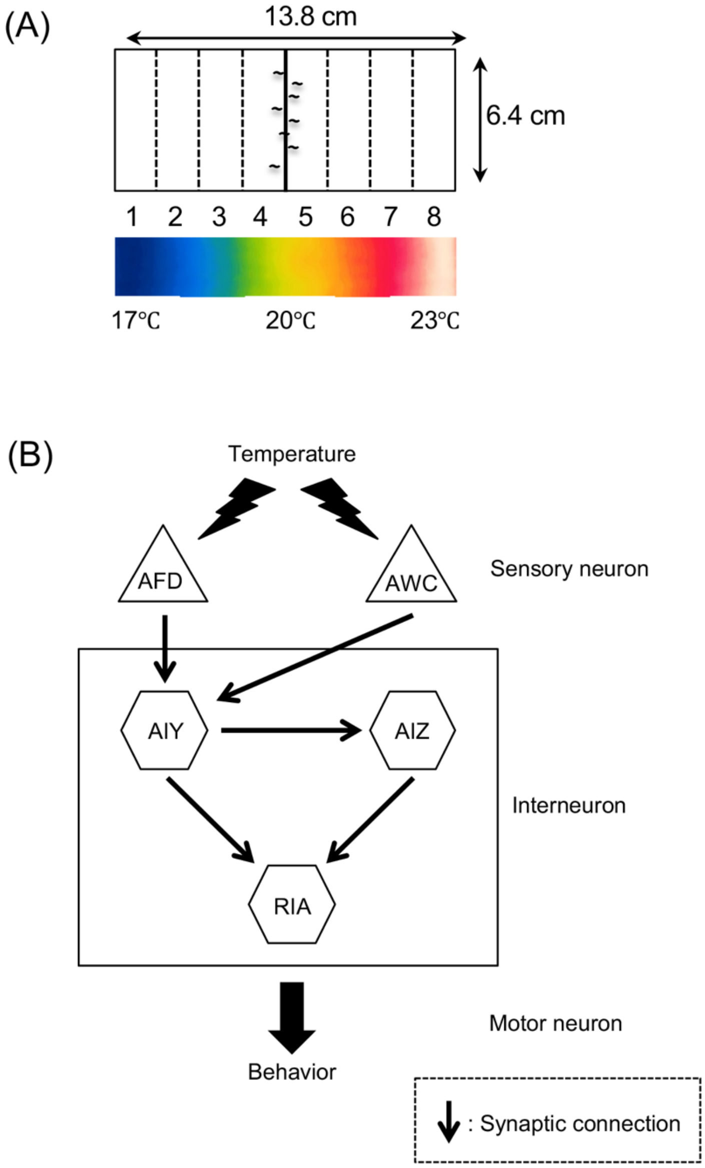

Fig. S3. Assay plate and neural circuit for thermotaxis behaviors

(A) Scheme of a plate with linear thermal gradient for thermotaxis assay. Plate area was divided into eight subregions along the gradient with score 1 to 8. A photo image by a thermal camera is shown below. (B) Neural circuit model for thermotaxis behaviors. Temperature is sensed by AFD and AWC thermosensory neurons. Activated AIY interneuron accelerates movement to a temperature higher than pre-cultivation temperature, whereas activated AIZ interneurons accelerate movement to a temperature lower than pre-cultivation temperature. The RIA interneuron integrates both thermal signals. Arrows indicate neural connections.

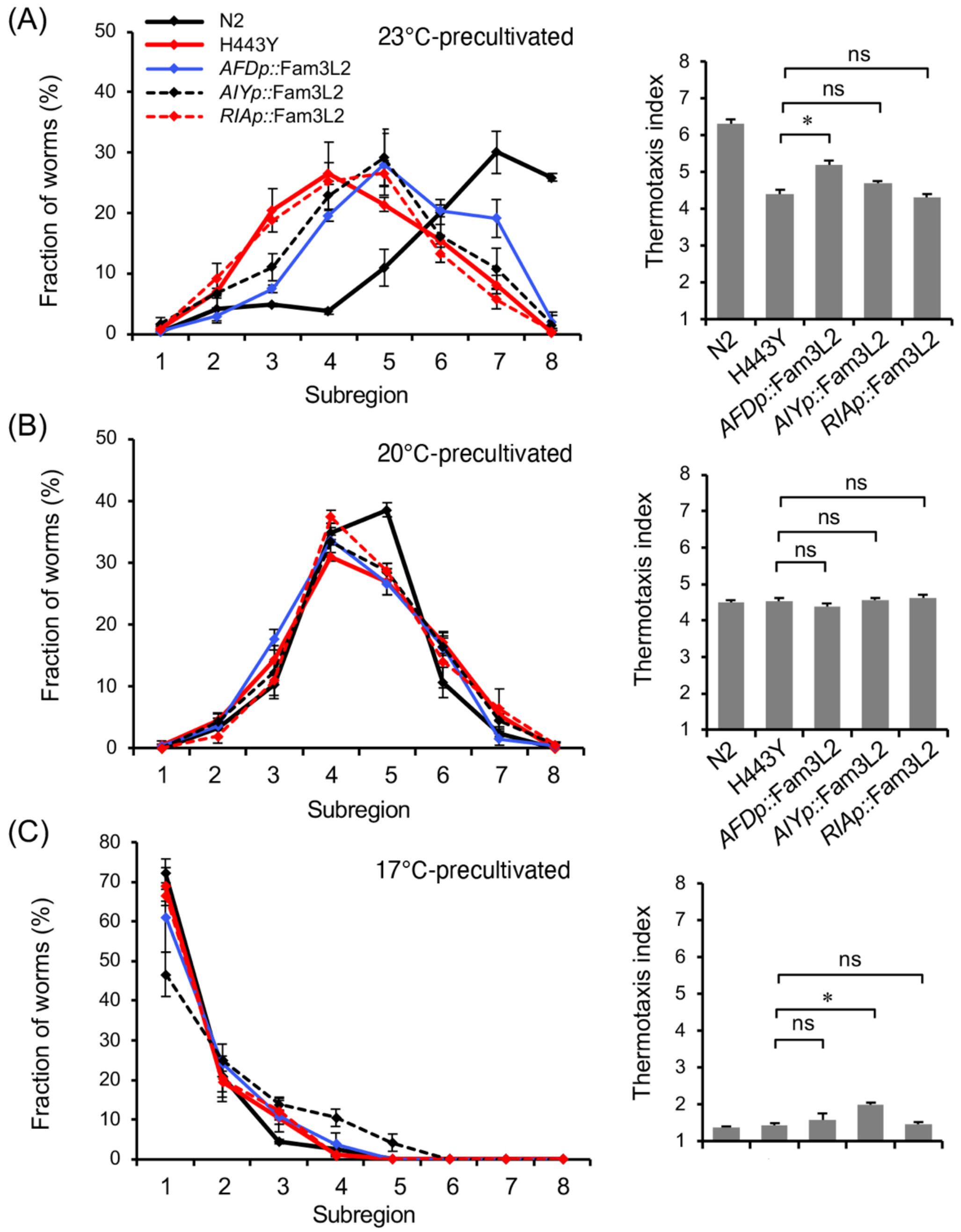

Fig. S4. Fam3L2 can rescue thermotaxis defect of H443Y-mutant worms

H443Y-mutant worms were injected with *gcy-18*, *ttx-3*, and *glr-3* promoter-fused cDNA for polypeptides corresponding to Fam3L2 domains (*AFDp::*Fam3L2, *AIYp::*Fam3L2, and *RIAp::*Fam3L2, respectively). Thermophilic (A), isothermal (B), and cryophilic (C) migrations were assessed. Line graphs show the percentage relative distribution of worms in each subregion. Thermotaxis index scores are shown in a bar graph. Data are means ± SEM; n = 3 for each genotype. *$P < 0.05$ and no significant difference (ns) according to ANOVA with Tukey's *post-hoc* test.

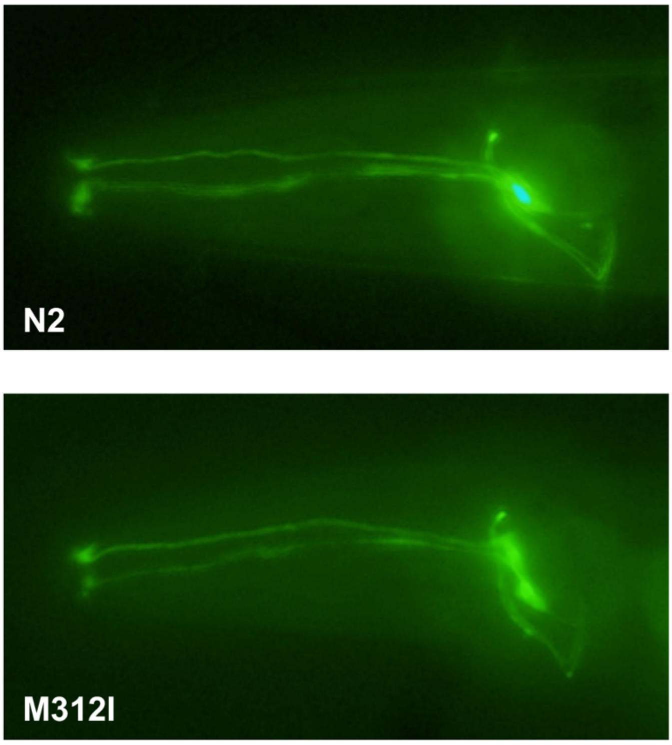

Fig. S5. Morphology of AFD neurons

Expression of a stably integrated *gcy-8::gfp* transgene in wild-type N2 (A) and M312I mutant (B) worms. Anterior at left in fluorescence images.

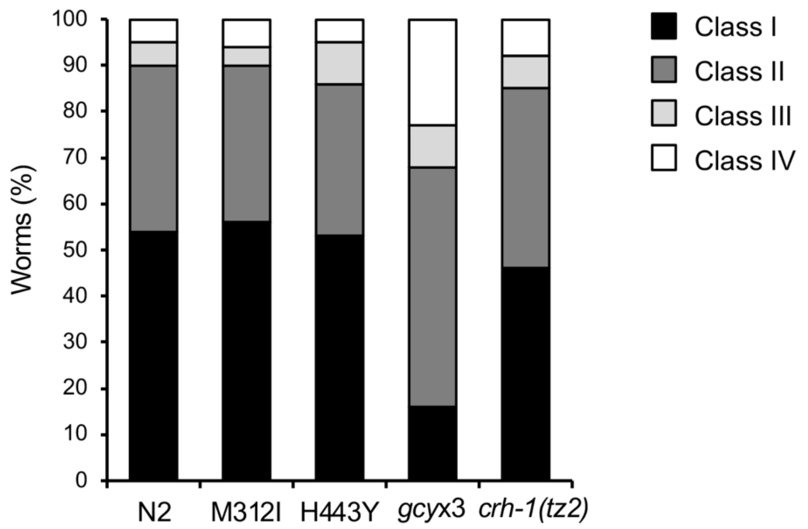

Fig. S6. Head avoidance from noxious temperature

Worms were exposed to noxious heat using an electronically heated metal tip. The heat stimulus was presented in front of the worm, and the initial response was classified according to a previous study (32) as follows: class I, rapid reflexive withdrawal, backing for at least one body length followed by a heading change; class II, rapid reflexive withdrawal but only a little backing; class III, slow backing; class IV, no response. The responses of 100 worms were recorded for each genotype. The data presented represent the percentage of each class.

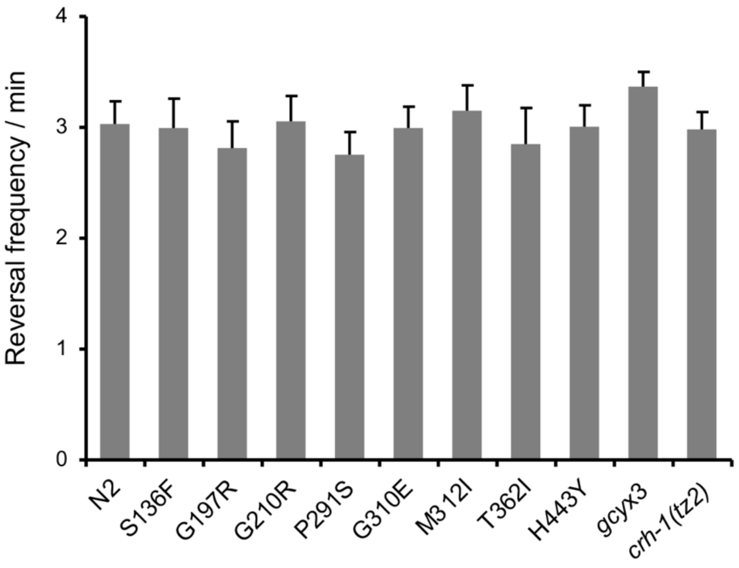

Fig. S7. Reversal behavior assay

Well-fed first day adult worms were transferred from growth plates to standard agar plates with no food. Any change from forward to backward movement was scored as a spontaneous reversal, and these changes were counted for 5 min. 20 worms were tested for each genotype. Data represent the number of spontaneous reversals per worm per min (means + SEM). There is no statistical difference between wild-type N2 and every mutant according to ANOVA with Tukey's *post-hoc* test.

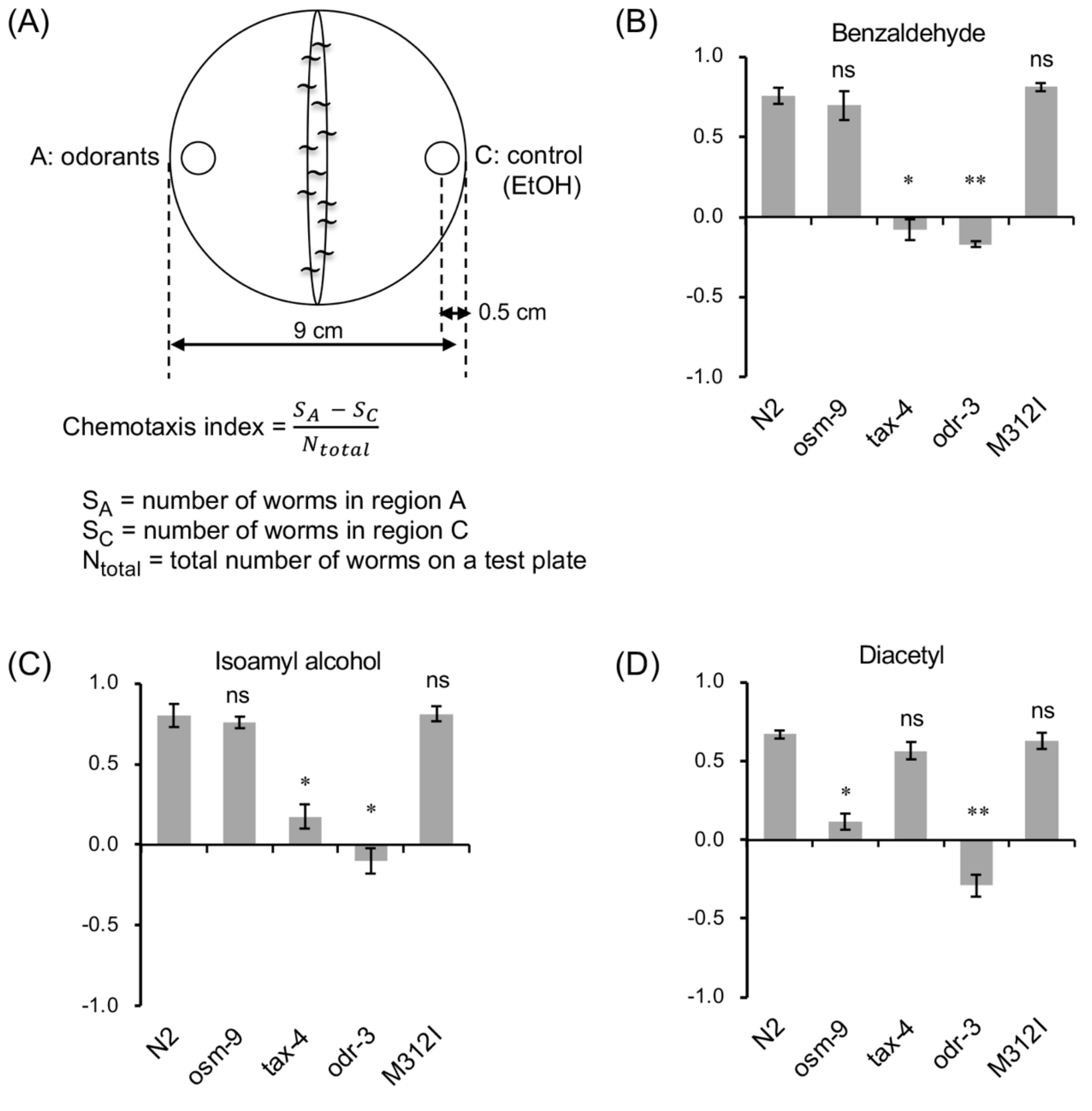

Fig. S8. Chemotaxis assay for M312I mutants

(A) Approximately 200 washed adult worms were placed near the center of a 9-cm assay plate with the attractant at one end of the plate and a control counter attractant at the opposite end of the plate. After 60 min, the numbers of animals in the attractant area and the control area were counted. A chemotaxis index was calculated based on the enrichment of animals at the attractant. (B-D) Odorant responses of wild-type (N2), *osm-9* mutant, *tax-4* mutant, *odr-3* mutant, and M312I-mutant worms. Expression of *osm-9* in AWC, *tax-4* in AWA, and *odr-3* in AWC and AWA neurons are required for odorant sensations, and *osm-9*, *tax-4*, and *odr-3* mutants are representative mutants with chemotaxis defects. Dilution of odorants was 1:200 for benzaldehyde (B), 1:100 for isoamyl alcohol (C), 1:1000 for diacetyl (D) with ethanol. Data are means ± SEM; n = 3 for each genotype. **P* < 0.05 and no significant difference (ns) versus N2 according to ANOVA with Tukey's *post-hoc* test.

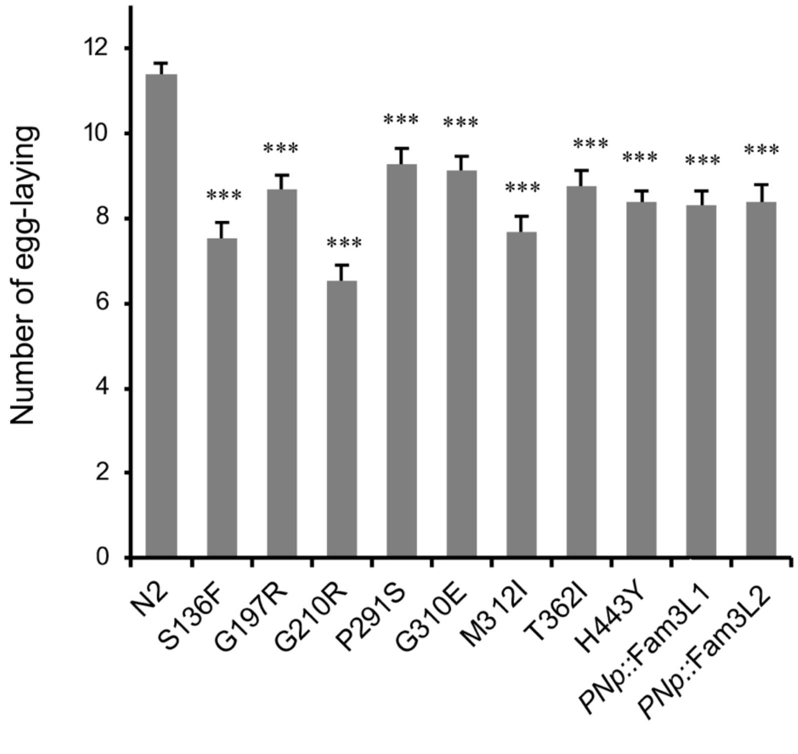

Fig. S9. Egg-laying behavior assay

Worms were picked at the early L4 stage and cultured at 20℃ before experiments. During the experiment, worms were allowed to lay eggs for 3 h on an NGM plate seeded with *E. coli* OP50-1. The number of laid eggs was counted using a standard dissection stereomicroscope. Data represent the number of egg-laying per worm per hour (means + SEM, n = 50 for each genotype). ****P* < 0.001 versus N2 according to ANOVA with Tukey's *post-hoc* test.

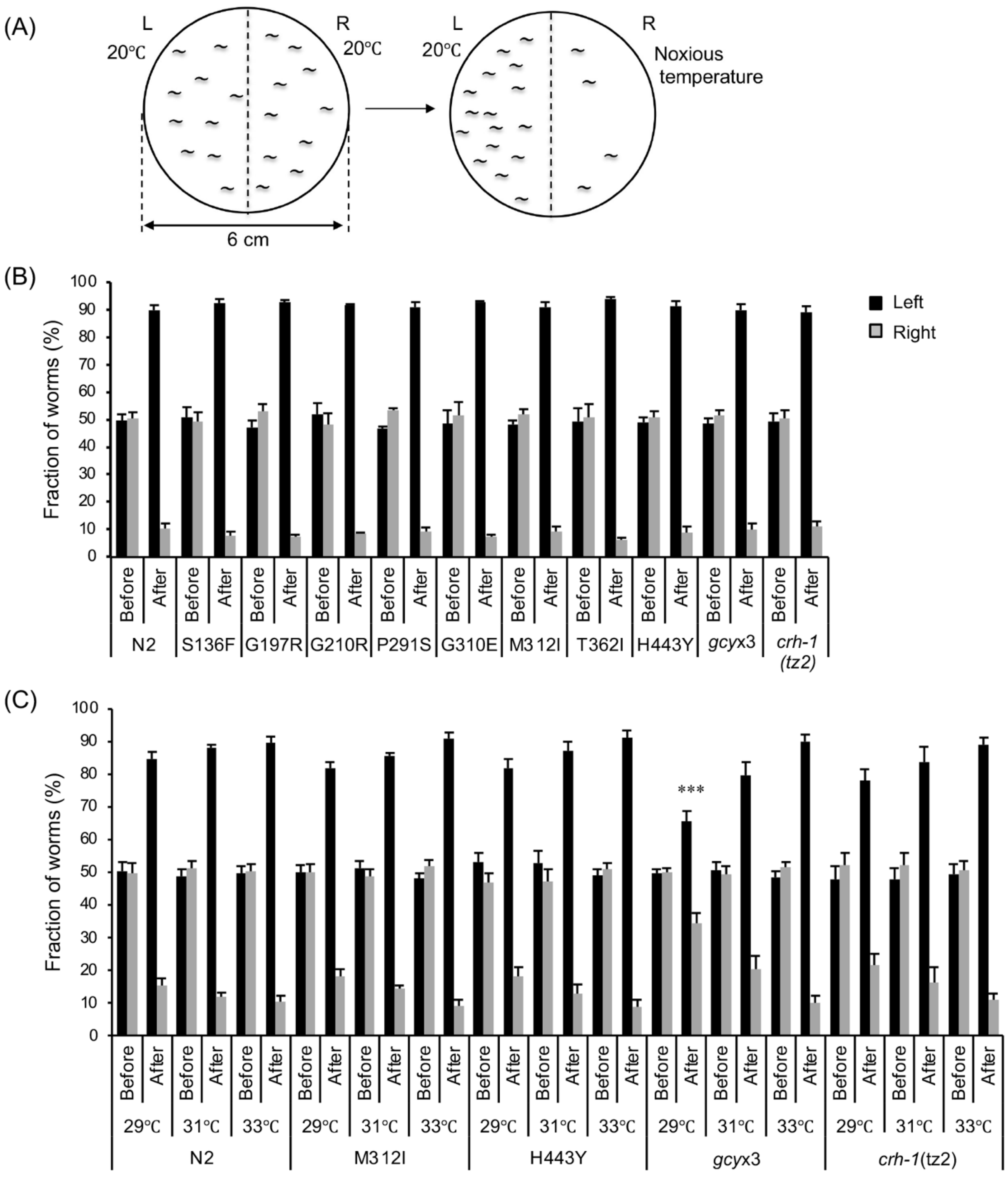

Fig. S10. Escape from higher cultivation temperature

(A) Well-fed first day adult worms were transferred to 6-cm agar plates with food and cultured at 20℃ for 60 min. The left half of the surface of the assay plate was then heated to a noxious temperature of 33℃ (B) and of 29℃, 31℃, or 33℃ (C) using a Peltier thermocontroller. Before and 60 min after heating, the number of worms on the right and left surfaces was counted. Assays were repeated three times for each genotype. Data are means + SEM. ****P* < 0.001 versus N2 according to ANOVA with Tukey's *post-hoc* test.

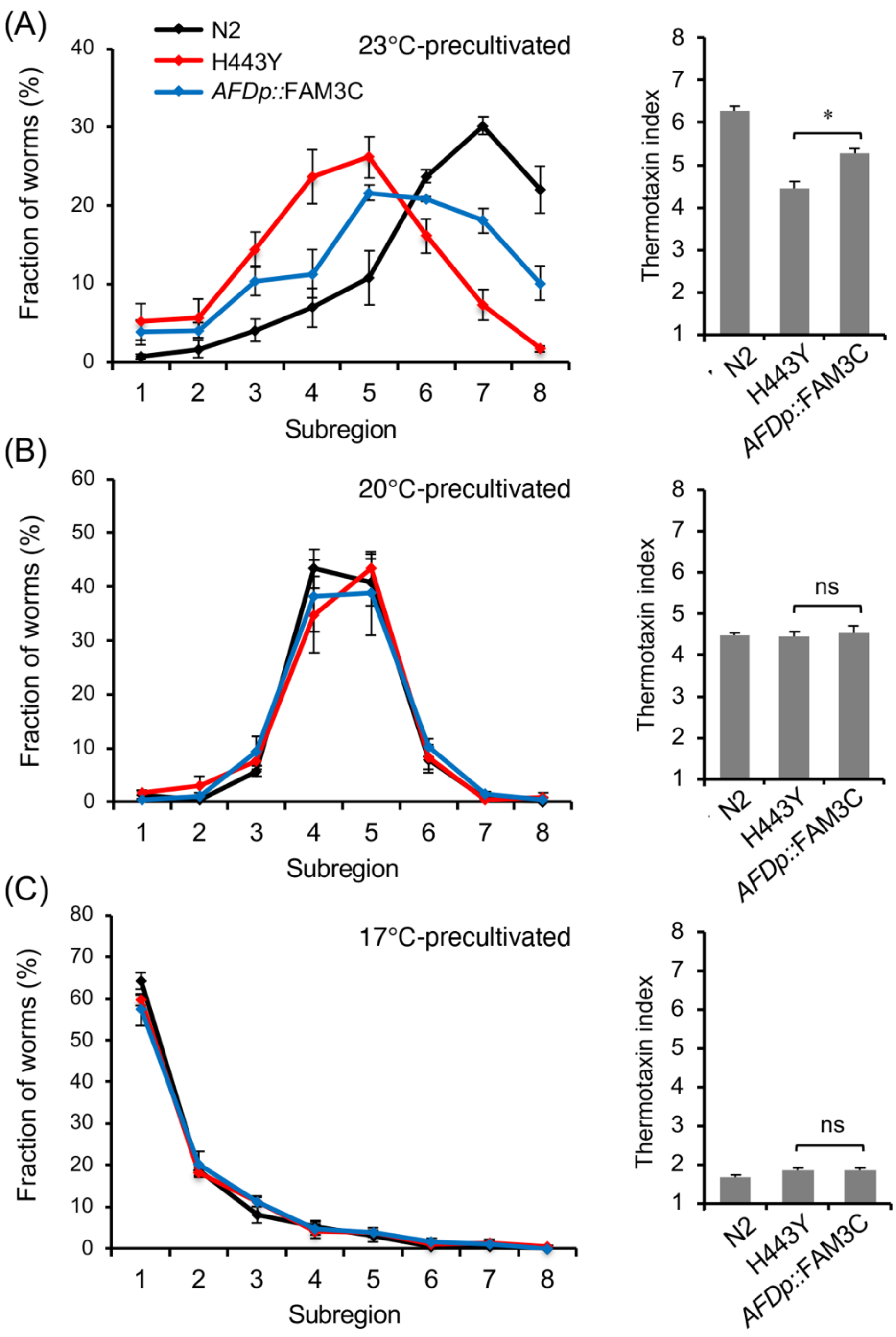

Fig. S11. Human FAM3C can rescue thermotaxis defect of H443Y-mutant worms

H443Y-mutant worms were injected with *gcy-18* promoter-fused codon-optimized cDNA for human FAM3C (*AFDp::*FAM3C). Thermophilic (A), isothermal (B), and cryophilic (C) migrations were assessed. Line graphs show the percentage relative distribution of worms in each subregion. Thermotaxis index scores are shown in a bar graph. Data are means ± SEM; n = 3 for each genotype. *$P < 0.05$ and no significant difference (ns) according to ANOVA with Tukey's *post-hoc* test.

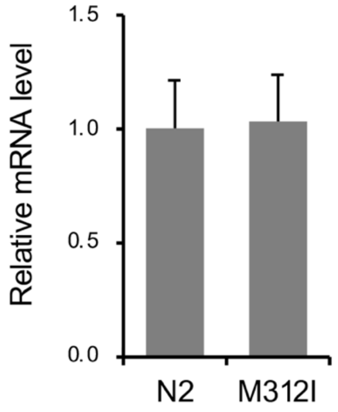

Fig. S12. The HSF-1 mRNA level in M312I mutants is equivalent to that of N2

Total RNA was extracted from 2-day-old wild-type N2 and M312I-mutant worms cultured on seeded plates. The HSF-1 mRNA level was measured by quantitative RT-PCR. The AMA-1 mRNA served as an internal control. Means + SEM; n = 4. No significant difference according to Student's *t*-test.

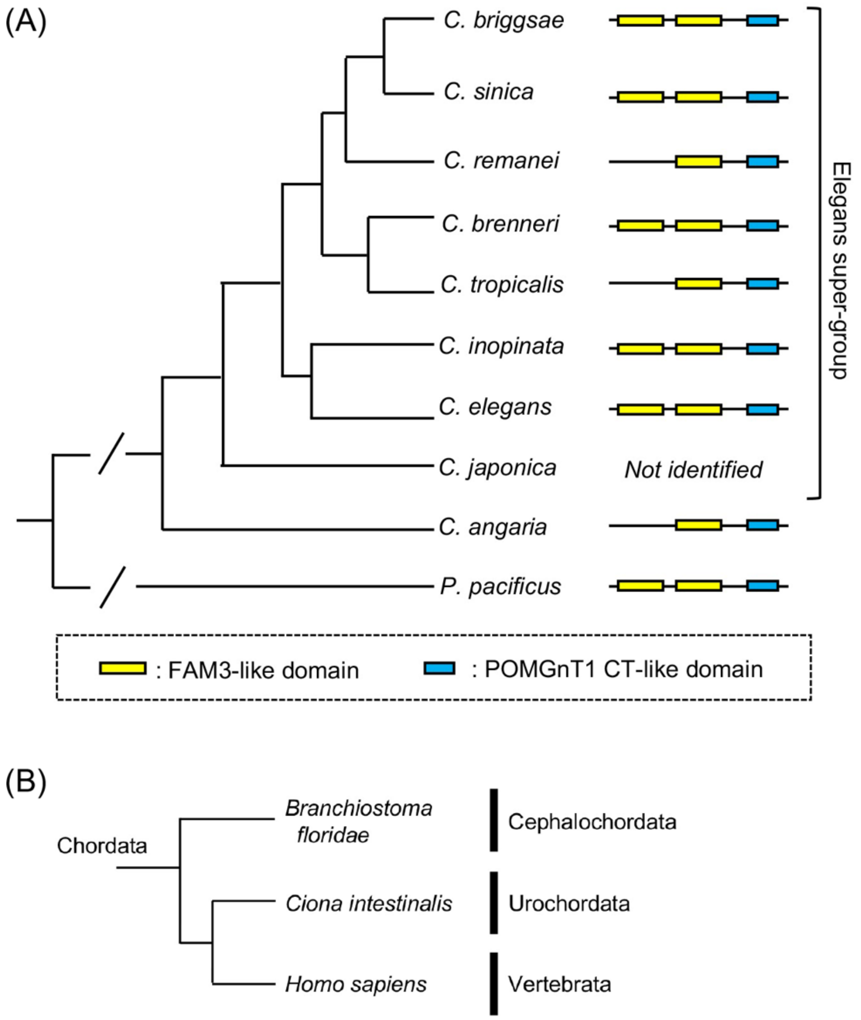

Fig. S13. Phylogenic trees.

(A) Phylogeny and *famp-1* orthologs of *Caenorhabditis* species. (B) Phylogenic tree of the phylum Chordata.
